# Evaluation of the Effects of Papain on *Schistosoma mansoni*: Miracidial Infection Capacity, Infection Prevalence, Cercarial Shedding and Molecular Changes in *Biomphalaria alexandrina*

**DOI:** 10.1007/s11686-024-00898-9

**Published:** 2024-08-27

**Authors:** Amina M. Ibrahim, Sami M. Nasr

**Affiliations:** 1 Medical Malacology Department, Theodor Bilharz Research Institute, P.O:11635, Imbaba, Giza Egypt; 2 Biochemistry, Molecular Biology and Medicinal chemistry Department, Theodor Bilharz Research Institute, Giza, Egypt; 3 School of Biotechnology, Badr University in Cairo, Badr City, Cairo 11829 Egypt

**Keywords:** Papain, *Biomphalaria alexandrina*, *Schistosoma mansoni*, Host-parasite interaction, Genotoxicity

## Abstract

**Purpose:**

The aim of the present study is to assess the molluscicidal, larvicidal and genotoxicological activities of papain and how it can affect the host-parasite interactions.

**Methods:**

Toxicity of papain on snails by making series of concentrations to calculate LC_50_, and then study its larvicide effect on the free larval stages of *S. mansoni* and infection rate of snails.

**Results:**

Papain has a molluscicidal activity on adult snails of *Biomphalaria alexandrina* with a lethal concentration LC_50_ equals to 43.1 mg/L. In addition, it has activity on miracidia with half Lethal time (LT_50_) of 16.11 min., and on cercariae with 12.1 min. compared to control ones. The sub lethal concentration LC_10_ and LC_25_ (6.9 or 24.1 mg/L, respectively) decreased the survival rate of snails at the first cercarial shedding, the rate of infection, the average total number of cercariae per snail, the shedding period and the life span of snails, while the prepatent period was significantly increased than the control ones. The morphological alterations in cercariae after exposure to papain were occurred where the cercariae lacked motility and some had a dark tail with complete detachment of head and tail. Compared to the control group, the levels of cytochrome oxidase subunit I (COI) and (ND1) genes significantly decreased in snails after exposure to papain.

**Conclusions:**

Papain could be used as a potential molluscicide for elimination of schistosomiasis and decrease its transmission and deterioration of host-parasite interaction.

## Introduction

Schistosomiasis is a neglected helminthic disease that is caused by trematodes of the genus *Schistosoma* in poor and undeveloped countries [1–3]. Estimates showed that more than 236.60 million people worldwide needed preventive treatment for schistosomiasis by *Schistosoma mansoni* [4–7]. The World Health Organization has established road map guidelines for the elimination of schistosomiasis based on mass drug treatment by praziquantel [8–10] and snail control [11, 12]. *Biomphalaria* snails are freshwater gastropods that are responsible for the transmission of *S. mansoni* [13, 14]. In this host, the miracidia penetrate its tissues and develop to cercariae (the infective stage) that emerge from snails and search for the final host to complete their life cycle [15, 16]. To control schistosomiasis, the life cycle could be cut at the snail stage [4]. The effective molluscicide should be biodegradable and nontoxic to non-target organisms like fish [17, 18].

Niclosamide is the only drug approved by WHO to eradicate snails of schistosomiasis [19, 20], but it is toxic to the non-target aquatic species besides the high cost and the pollution that resulted from its use [2, 16, 21]. Many investigations were evolved to study the effects of different plant molluscicides on the biological aspects of the snails and the larval stages of *S. mansoni* [4, 22–24].

Plants and their secondary metabolites can be used as potential molluscicidal, herbicidal, antimicrobial and helminthicidal agents [18, 25–28]. The main reason for using plant molluscicides is they are cheap, biodegradable, easily handled and can provide a good income for poor farmers in undeveloped countries [23, 29–32].

*Carica papaya* L. (Caricaceae) is widely cultivated for food and has several industrial uses [33]. It is known to have antischistosomal and molluscicidal activities due to the presence of papain [34, 35]. Phytochemical screening of the ethanol extract of *C. papaya* seeds revealed the presence of polyphenols and glycosides, along with trace amounts of alkaloids, saponins, and flavonoids [36]. Papain is a purified protein extracted from the latex of the unripe papaya, is widely used in folk medicine [37]. It is a predominant enzyme that has the ability to cause a genotoxicity or mutagenicity in DNA [33]. Also, papain is a potent molluscicide against the harmful snail *Lymnaea acuminata*, the intermediate host for the liver fluke *Fasciola gigantica* [38]. Another study stated that the ethanolic extract of *C. papaya* seed (LC_50_ at 24 h: 53.38 mg/l) could be used as potent molluscicide against *L. acuminata* since this concentration is not toxic for *Colisa fasciatus* fish which is living in the same habitat with the snail [39]. The methanolic extract of *C. papaya* could be used as an eco-friendly alternate molluscicide for *B. alexandrina* snails with safety effects on *D. magna* with no mortality percentages of *D. magna* during the first 12 h of the exposure to 138.5 mg/l [31].

Therefore, the main objectives of this study were (i) to study papain larvicidal effects on the free larval stages of *S. mansoni*, (ii) to determine how papain can affect host-parasite interaction by specifying the prevalence of snail infection, the length of the prepatent and patent periods, the shedding of cercariae and, also, the survival rate and lifespan of exposed snails, and (iii) to elucidate its molluscicidal effect on DNA of exposed *B. alexandrina* snails by a real-time PCR.

## Materials and Methods

### Snails and Parasite

#### Snails

Adult *B. alexandrina* (9–11 mm) were obtained from a reared lab colony in Medical Malacology Laboratory, Theodor Bilharz Research Institute (TBRI), Giza, Egypt.

#### Schistosoma mansoni Miracidia

*Schistosoma mansoni* eggs were obtained from the Schistosome Biological Supply Center (SBSC), Theodor Bilharz Research Institute, Giza, Egypt (SBSC/TBRI). This strain originated from Egypt and was obtained from Giza Governorate and has been maintained in albino mice *Mus musculus* CD1 strain. Eggs were left in a clean dechlorinated tap water (24 ± 1 °C) for hatching under a desk lamp light. Freshly hatched miracidia were pipetted in clean petri-dishes for further experiments [40].

#### Cercariae

*Schistosoma mansoni* cercariae were obtained from the SBSC/TBRI, Giza, Egypt. It was shed under illumination from the infected *B. alexandrina* snails and they were used for cercaricidal activity immediately after they were shed from these snails.

#### Snail Maintenance

Adult snails were reared in 15 × 24 × 10 cm plastic aquaria (4 L capacity) and provided with dechlorinated tap water which was changed each 3 days. Snails were fed on lettuce, blue green algae (*Nostoc muscorum*) and tetramine (fish food) (10 snails/ L). The temperature of aquaria was adjusted to 25 ± 2 °C, pH: 7 ± 0.2, with a photoperiodicity of 12 h light/12 h dark [41]. The egg masses were collected by fine forceps from small foam pieces that were placed on the water surface of the aquaria [4]. Egg masses were transferred to clean smaller plastic aquaria of 2 L capacity containing dechlorinated tap water and fed with blue green algae (*Nostoc muscorum*), tetramine and small pieces of CaCO_3_ [6]. After reaching a 4–5 mm shell diameter, they were subjected to the infection with miracidia.

### Papain

Papain from *Carica papaya* latex (lyophilized powder, 23 kDa, Alfa Aesar) was obtained from ThermoFisher (Kandel) GmbH- Erienbachweg 2-76870 Kandel, Germany. To calculate LC_50_ and LC_90_, serial dilutions of papain (10, 15, 25, 50, 75, and 100 mg/l) were made with dechlorinated tap water.

### Snail Infection

Three replicates, each of 20 lab-bred *B. alexandrina* snails (4–5 mm), were exposed to newly hatched *S. mansoni* miracidia, 10 miracidia/ snail for 2 h under illumination and were maintained till cercarial emergence.

### Toxicity of Papain on Snails

#### Uninfected Adult Snails

Ten snails (diameter, 9–10 mm) were exposed to each concentration tested in a plastic aquarium [42] and three replicates were performed. Control snails of the same size were maintained only in dechlorinated tap water and their behavior was assessed against that of their papain-exposed congeners under the same experimental conditions [43]. The duration of exposure to papain lasted 24 h and was followed by a recovery period (in dechlorinated water) for 24 h. Mortality percentages were analyzed by a probability analysis [44] to determine sublethal concentrations of the substance [45].

#### Infected Snails

Three replicates, each with 20 laboratory-reared *B. alexandrina* snails (4–5 mm), were exposed to newly hatched *S. mansoni* miracidia (10 miracidia/ snail) for 2 h under illumination and the sublethal concentrations LC_10_ or LC_25_ (6.9 or 24.1 mg/L) of papain for 24 h and placed in dechlorinated tap water afterwards. Three other replicates, each comprising 20 snails, were exposed to miracidia under the same conditions and kept only in dechlorinated tap water until cercariae emerged to constitute a positive control group.

##### Examination of the Infected Snails for the Cercarial Shedding

This examination starts from day 21 post miracidial exposure. Snails were examined individually for cercarial shedding in multi-dishes under artificial light for 2 h and 2 ml of dechlorinated tape water/snail. As soon as the initial shedding was observed, positive snails were separated individually in plastic cups and examined once a week till snails’ death. The emerged cercariae/ snail were pipetted to a small Petri dish, fixed in Bouin’s solution and counted under a stereomicroscope to count the total cercariae/ snail.

- The snail’s infection rate was calculated at the end of experiment by dividing number of shedding and positive snails on the number of survived snails at first shedding X100 [46].
- The survival rate was calculated by dividing the number of snails at first shedding by the total number of exposed snails [47].
- Mean prepatent period, mean length of shedding, and mean life- span of snails in each group of positive infection [48].

### Papain Toxicity on *Schistosome*- free Larvae

Miracidicidal, cercaricidal activity and their lethal time (LT_50_, _90_, and _99_):

One hundred newly hatched miracidia/ 5 ml dechlorinated water were placed in a sterilized petri dish with 5 ml of LC_50_ concentration of papain (43.1 mg/L). In addition, freshly shed 100 cercariae/ 5 ml water were placed with the same concentration of papain. As well as, the control groups were assessed side by side as 100 miracidia or cercariae were kept in 10 ml of dechlorinated tap water [49]. Three replicates were used to detect the number of dead miracidia and cercariae with each concentration and control groups [50, 51]. Both larval stages were examined under a stereomicroscope to detect their morphology and motility after intervals of 10, 20, 30, 40, and 50 min. The stationary cercariae or miracidia were photographed by light microscope and they were considered as dead [13, 52]. Cercariae were considered dead when they stopped movement, sank down and when their tails were detached [51]. It was photographed by Automatic camera using Olympus System Microscope.

Lethal Time (LT_50_, _90_, and _99_) is the time of the death of 50%, 90% and 98% of *S. mansoni* larvae after exposure to the half lethal concentration LC_50_ (43.1 mg/l) of papain. Determination of the value is done by probit analysis [53].

### Molecular Changes in Papain-Exposed Adult Snails

Snails were exposed to the sublethal concentrations either LC_10_ 6.9 mg/L or LC_25_ 24.1 mg/L of papain for 24 h followed by 24 h recovery in dechlorinated water. Snails were dissected and the head foot part was used in Gene regulation assay by quantitative PCR as reported by [13]. They used two primers to measure changes in *B. alexandrina* cytochrome oxidase subunit I (COI) and *B. alexandrina* NADH dehydrogenase subunit 1 (ND1) genes. Both genes were quantified using the StepOne Real-Time PCR System (Applied Biosystems, California, USA) duplicated real time runs [54].

*B. alexandrina* cytochrome oxidase subunit I (COI) product length is 449 bp.

Forward primer (GGTACTACTCTTGTTTTGATAGATG) Tm = 52 and.

Reverse primer (GCTGTAACCAACACAGATCATAC) Tm = 54.

*B. alexandrina* NADH dehydrogenase subunit 1 (ND1).

Forward primer (GGGATTCTGCAACCATTTGC).

Reverse primer (TTTCTGCTAATGTTGTTGTAAATCACAC) Tm = 55 for both and Product length is 439. The slides were coded independently and scored blindly.

### Statistical Analyses

The half-lethal concentration values were defined using the Probit facility [44]. Student’s t-test was used to compare the means of the exposed and control groups [55]. Infection rate of every snail group was compared with control using the chi-square (χ2) test [56]. Significant differences were considered at *p* ≤ 0.05. Data were expressed as mean ± standard error of the mean (SEM). Data normality has been checked using the Shapiro-Wilk test [57].

## Results

**Fig. 1:**
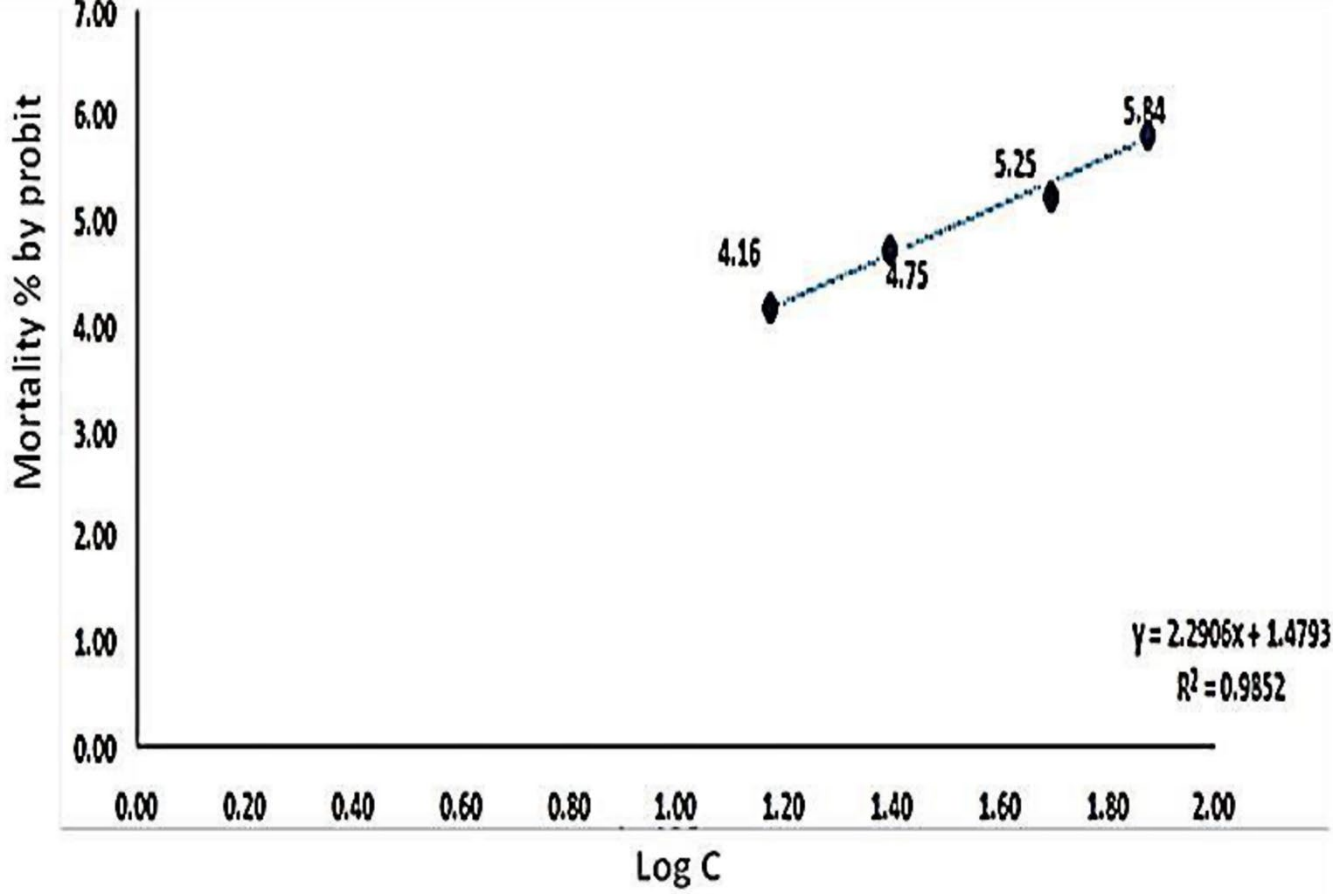
Mortality % by probit of *Biomphalaria alexandrina* snails after 24 h exposure to different concentrations of papin (with Good Curve fitting)

The present results showed that papain has a molluscicidal activity against adult *B. alexandrina* snails after 24 h of exposure (Table 1, and Fig. 1) with a median lethal concentration (LC_50_) 43.1 mg/l.

**Table 1:** Shows the molluscicidal activity of papain against adult *Biomphalaria alexandrina* snails (24 h exposure)

Concentration (mg/l)	LC_10_ (mg/l)	LC_25_ (mg/l)	LC_50_ (mg/l)	Confidence interval	LC_90_ (mg/l)	slope
Papain	6.9	24.1	43.1	25.4- 65.01	79.1	1.4

### Snail’s Infection Parameters

**Fig. 2:**
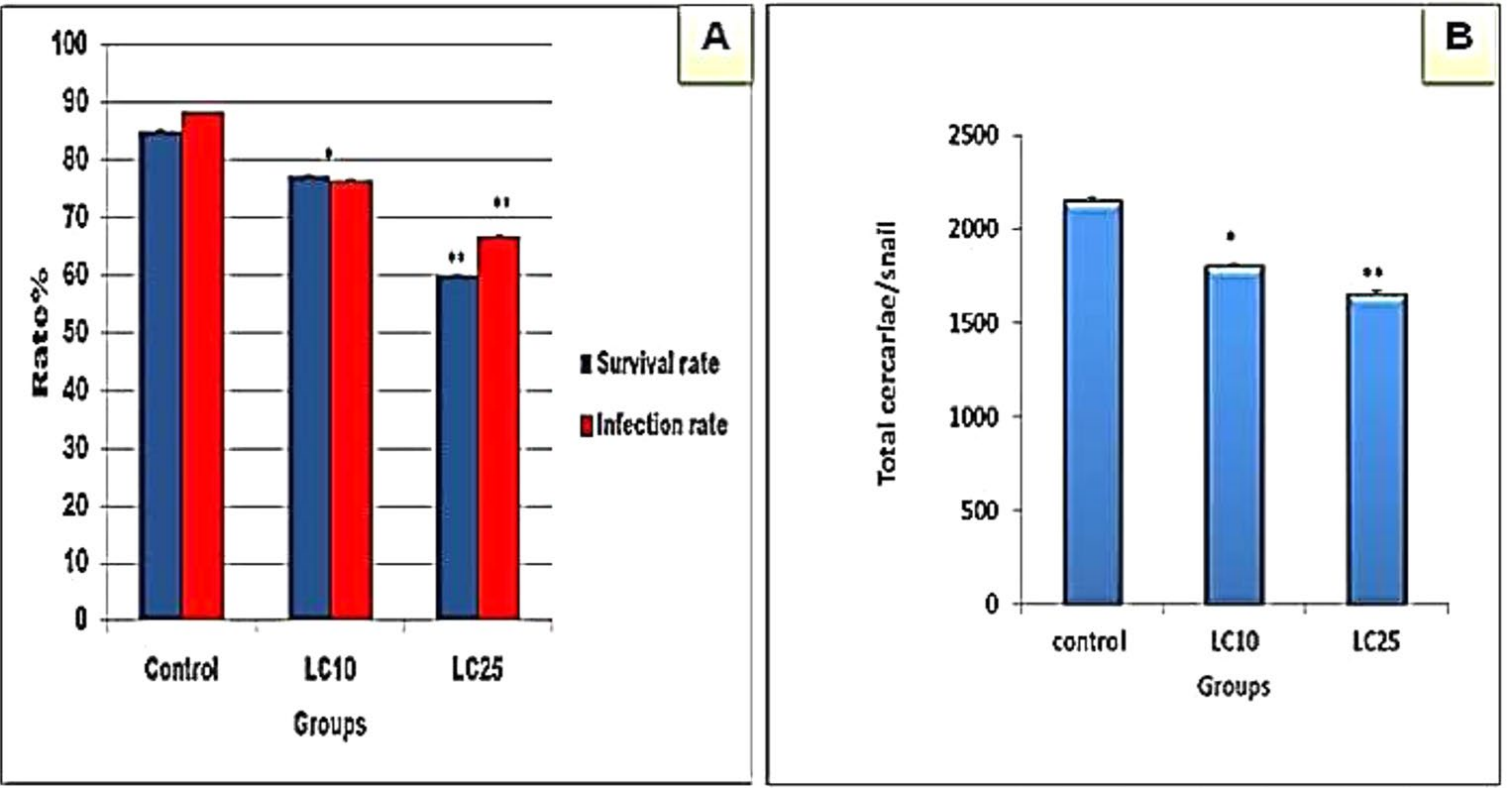
Effect of the sub lethal concentration LC_10_ or LC_25_ (6.9 or 24.1 mg/l) of papain on: (**A**) the survival rate at 1st shedding, infection rate of *B. alexandrina* and; (**B**) the total number of cercariae/ snail. * Significant at *p* < 0.05, ** significant at *p* < 0.01

The present results showed that after exposure to the sub lethal concentration LC_10_ or LC_25_ (6.9 or 24.1 mg/l) of papain for 24 h and exposure to *S. mansoni* miracidia, both the survival rate at first cercarial shedding and the infection rates were significantly decreased (Fig. 2, A). Also, the present result confirmed that the mean total number of cercariae that were shed by each snail was significantly (*p* < 0.01) decreased than the control ones (Fig. 2, B).

**Fig. 3:**
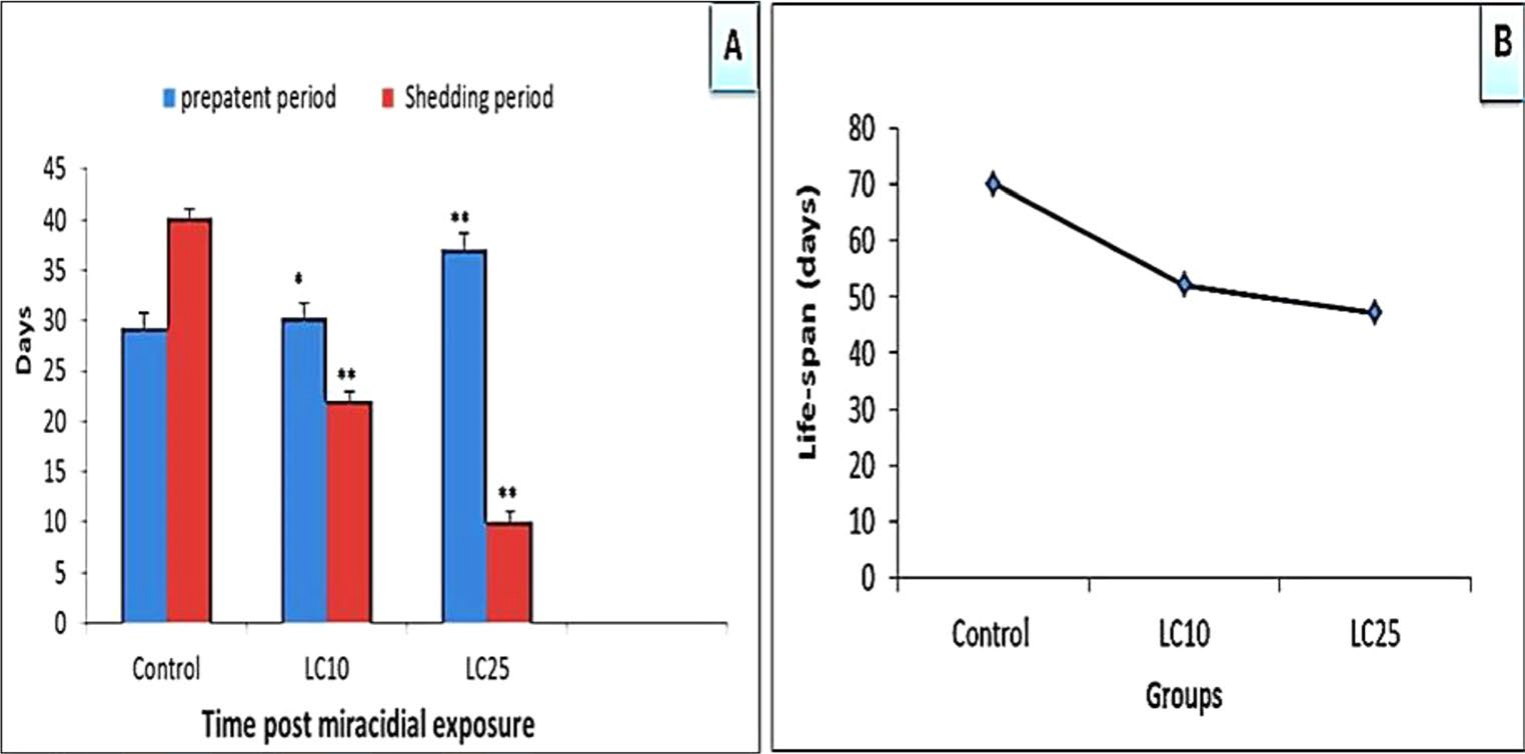
The effect of the sub lethal concentration LC_10_ or LC_25_ (6.9 or 24.1 mg/l) of papain on the pre-patent period, shedding duration post miracidial exposure and the life span of these snails. * Significant at *p* < 0.05, ** significant at *p* < 0.01

In *S. mansoni*-infected snails, the prepatent period significantly increased (*p* < 0.01) after exposure to a LC_10_ (at 6.9 mg/l) or LC_25_ (at 24.1 mg/l) of papain for 24 h, whereas the shedding period was significantly lower (*p* < 0.01) than that observed in controls infected and not exposed to papain (Fig. 3, A). The lifespan of infected snails exposed to papain also significantly decreased (*p* < 0.01) compared to infected controls and was dependent on the concentration of the product used (Fig. 3, B).

### Larvicidal Effect of LC_50_ (43.1 mg/l) of Papain

**Fig. 4:**
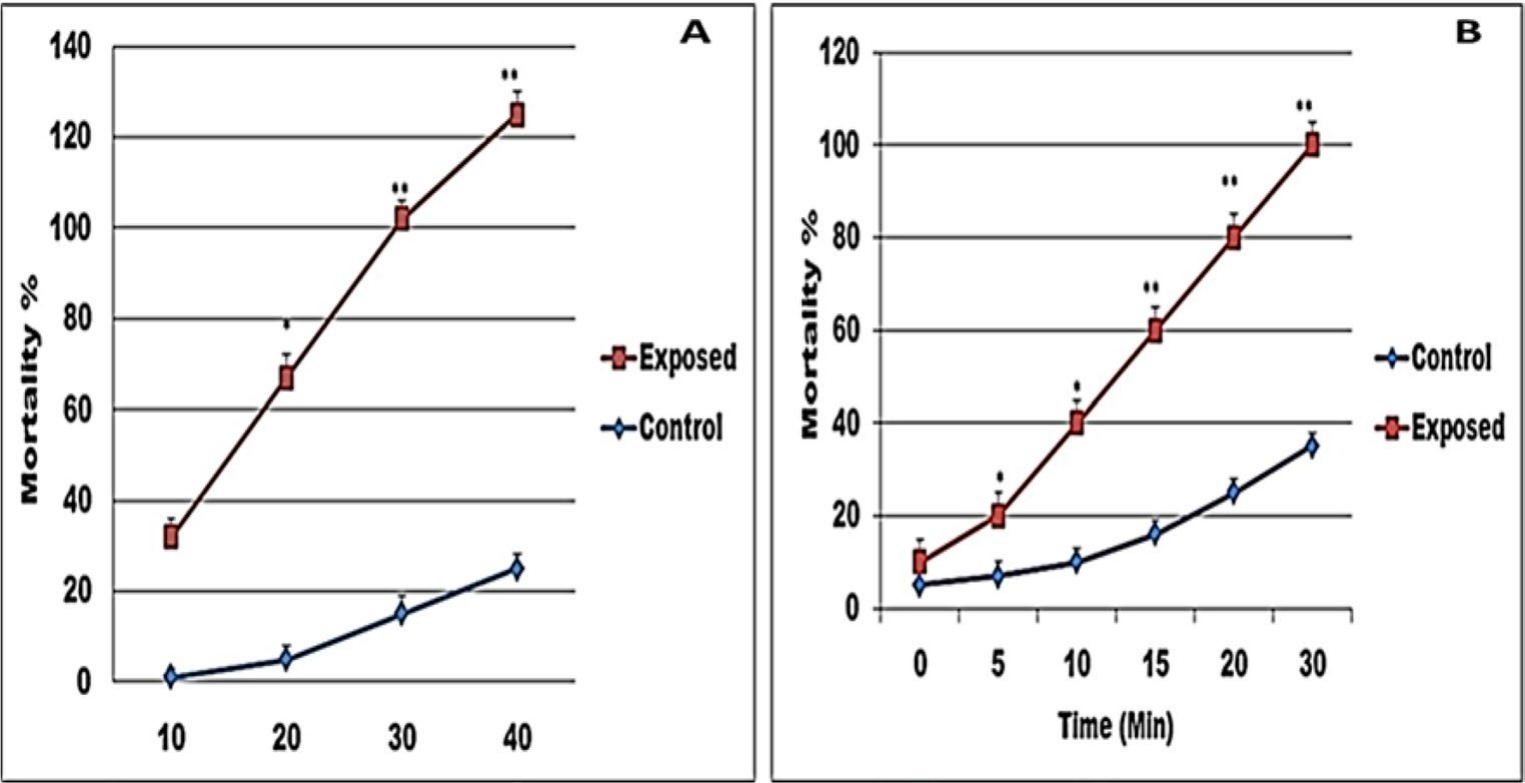
Shows the effect of the sub lethal concentration LC_50_ (43.1 mg/l) of papain on *S. mansoni* larvae; (**A**) miracidia and (**B**) cercariae

The present results showed that the sub lethal concentration LC_50_ (43.1 mg/l) of Papain has miracidial activity where all miracidia died after 40 min. Also, it has cercaricidal activity, where all cercariae died after 30 min of exposure compared to control ones (Fig. 4).

**Fig. 5:**
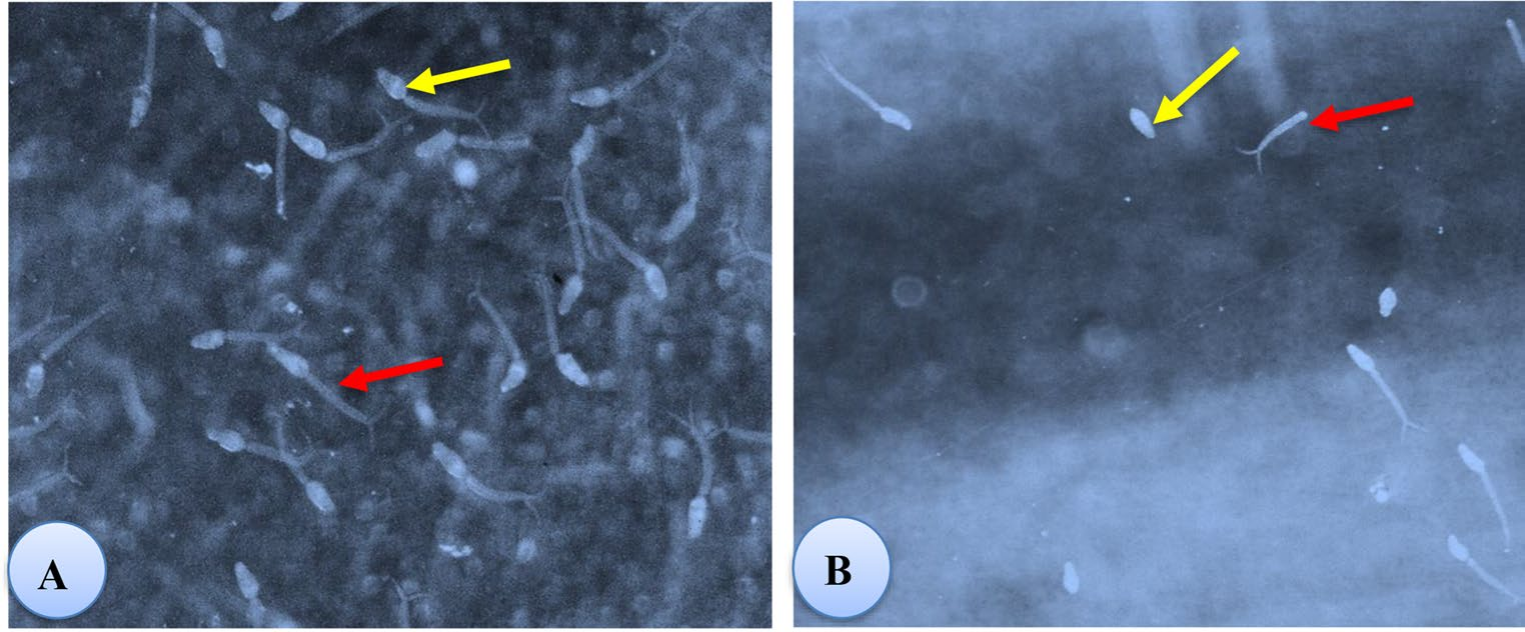
Morphological alterations in *Schistosoma mansoni* cercariae after exposure to a LC_50_ of papain (at 43.1 mg/l): control cercaria (**A**), dead cercaria with complete detachment of the head and tail (**B**)

The present results showed some morphological alterations in cercariae were observed after exposure to the sub lethal concentration LC_50_ (43.1 mg/l) of papain, where they lack their motility and this is considered as the death of the larval stages. Also, cercariae showed darkness and swollen head, when exposed to papain for 30 min there was a complete detachment of the head and tail (Fig. 5).

Regarding the half Lethal Time (LT50) for miracidia and cercariae, the present results showed that the sub lethal concentration LC_50_ (43.1 mg/l) of papain has affected the *S. mansoni* larvae lethal time, where the half Lethal Time (LT50) for miracidiae was 16.11 min, while for cercariae was 12.1 min (Table 2).

**Table 2:** Lethal Times (LT50, 90 and 99) of larval stages of *Schistosoma mansoni* against LC_50_ (43.1 mg/l) of papaiin

Concentration	Lethal time	Time (min)	95% Confidence Limit	
			Lower	Upper
Miracidia	LT_50_	16.11	14.04	17.8
	LT_90_	30.5	28.02	31.9
	LT_99_	42.4	40.01	44.01
Cercariae	LT_50_	12.1	11.1	13.2
	LT_90_	23.2	21.1	25.1
	LT_99_	32.2	30.1	34.1

**Fig. 6:**
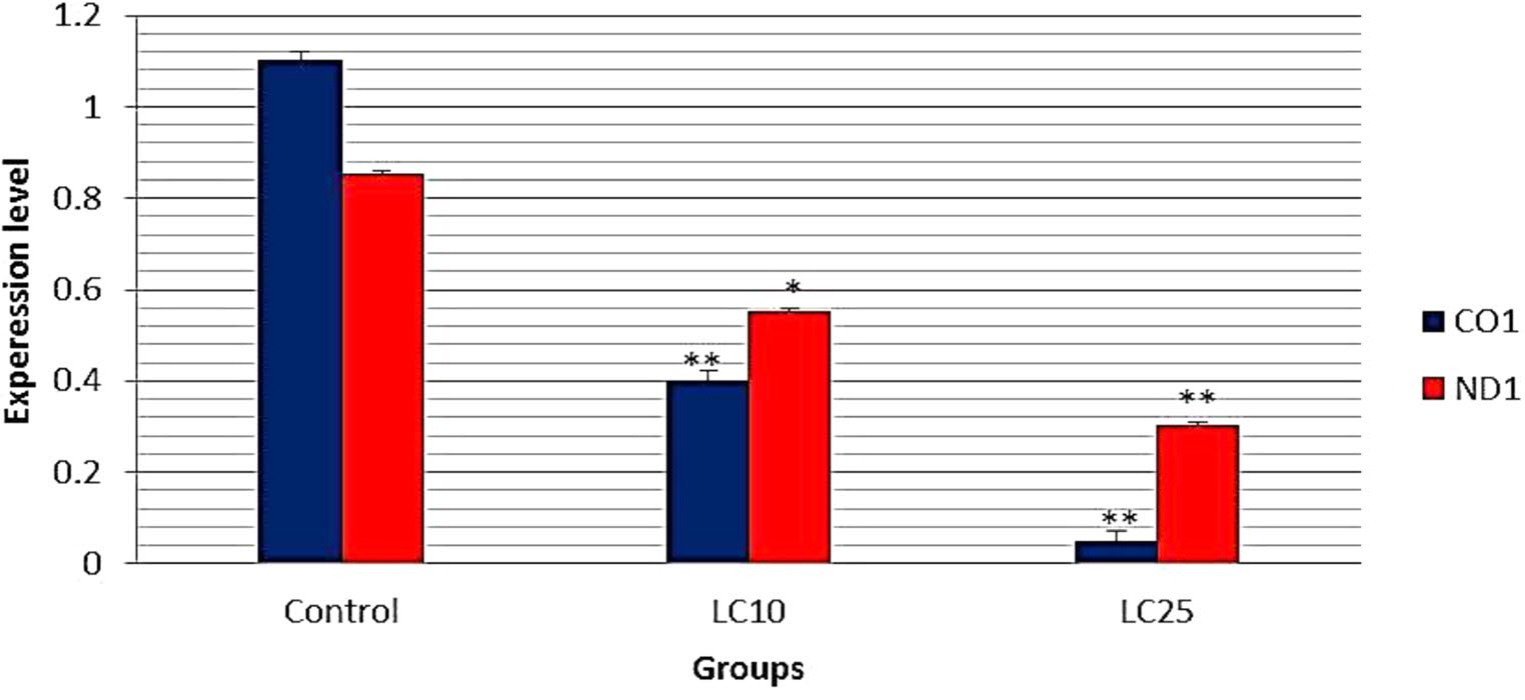
Relative quantification of *B. alexandrina* cytochrome oxidase subunit I (COI) and (ND1) genes after exposure to the sub lethal concentration LC_10_ or LC_25_ (6.9 or 24.1 mg/l) of papain. * Significant at *p* < 0.05, ** significant at *p* < 0.01

The present results showed that the expression level of cytochrome oxidase subunit I (COI) and (ND1) genes were significantly decreased (*p* < 0.01) in exposed snails than the control group and this reduction is concentration dependent (Fig. 6).

## Discussion

The present results showed that Papain has molluscicidal activity on adult *B. alexandrina* snails after 24 h with lethal concentration (LC_50_) was 43.1 mg/l. The molluscicidal activity of the lyophilized latex powder of *Carica papaya* (papain) was also confirmed on *Lymnaea acuminata* snail (LC_50_ at 96 h: 8.38 mg/l) and this toxicity is time and dose dependent [39]. Authors made another paper on the mechanism of the action of papain and how it affected the biological system, they found that papain (*C. papaya* latex) significantly inhibited the acetylcholinesterase (AChE), acid and alkaline phosphatase(ACP/ALP) activity in the nervous tissue of *L. acuminat*a [58]. In 2018, another study showed that snail fed on pellets containing papain (40% of 24 h LC_50_) resulted in significant decreases in protein, amino acids, DNA, RNA and AChE levels in the gonadal/nervous tissue of *Lymnaea acuminata* and this is might be the cause for snails death [59]. Also, the *C. papaya* ethanolic leaf extracts has molluscicidal activity on *B. globosus*, the snail intermediate hosts of schistosomiasis [60].

The present results showed that after exposure to the sub lethal concentration LC_10_ or LC_25_ (6.9 or 24.1 mg/l) of papain for 24 h and exposure to *S. mansoni* miracidiae, both the survival rate at first cercarial shedding and the infection rates, mean total number of cercariae per snail, the shedding period and the life span of snails were significantly decreased, while the prepatent period was significantly increased than the control ones in concentration dependent way. The time-dependent toxic effects of papain might be correlated to the snails’ uptake of the active components which will increase in their body with the increase in the exposure duration [61].

The interaction between mollusks and their trematode parasites is dynamic and in which the trematode is either destroyed and eliminated by the host snail defensive system [62, 63]. These decreases might be due to the excessive production of the inhibitory compound by the infected snails and further histopathological damages which might lead to the reduction in these parameters [64] or might due to a disruption in the snail metabolism due to exposure to *S. mansoni* [65, 66]. Also, Papain can be used in vaccination of CD-1 mice and hamsters against *S. mansoni* and *S. haematobium*, respectively, where it decreased the worm burden, egg load, and the viability of the parasite ova in the small intestine and liver [67].

The present results showed that the Papain sub lethal concentration LC_50_ (43.1 mg/l) has miracidial and cercaricidal activities. This might be due to the toxic effect of papain on the enzymatic activities of the miracidiae which would lead to their death and hence decreased the infection rate of snails by these miracidiae [68]. Similarly, [69] reported 100% mortality of cercariae and miracidiae after 30 min and 60 min exposure to the sublethal concentration of *Albizia anthelmintica* saponin (LC_50_ 17.6 ppm) and this resulted in a significant reduction of the survival and rate infection rate of the exposed snails. Authors reasoned these alterations to the high damages in the physiological and biological parameters of snails which made them unsuitable intermediate host for the parasite development [13]. These results were reflected on the reduction in all parameters of infection measured in the present study.

The present results showed some morphological alterations in cercariae after the exposure to papain, where they lack their motility and some has dark tail with complete detachment of head and tail. These results in good accordance with [51] who reported that the microscopic examination of cercariae exposed for 1 h to *Solanum nigrum* and *Callistemon citrinius* leaves extract where cercariae have a decrease in motility until motionless with shorten length. Some have abnormal shape, head separation from tail, and sometimes disintegration. Because of theses alterations, the measured infection parameters were decreased. Also, [70] reported miracicidicical and cercaricidal activities of *Nerium oleander* and *Tecoma stans* extract and correlated the mortality of these larvae to the presence of the bioactive secondary metabolites in their extracts [71]. Also, [72] confirmed the presence of the cercarial abnormalities after exposure of *S. mansoni* cercaria to different concentrations of *Solanum nigrum*, where, the cercariae showed swollen head, darkness of the tail and shortness, complete detachment of head and tail with loss of their contents [51].

The Lethal Time (LT) is a standard medium time measurement that can kill test animals. It was done to find out the time needed for LC_50_ concentration of papain [53]. The half Lethal Time (LT50) for miracidiae was 16.11 min, while for cercariae were 12.1 min compared to control ones. The causes of this mortality is due to the interference of papain with the protein biosynthesis process, RNA, DNA and AChE which would lead to the larval death [23, 58, 70].

Genotoxicological studies on DNA were considered as an effective tool for assessing the contamination of the aquatic ecosystems [6, 18, 73]. The main reason in this genotoxic effect is the increase in reactive oxygen species that responsible for these changes [74]. The present results showed that the expression of COI was significantly decreased compared to the expression of ND1in all groups. After exposure to the sub lethal concentration LC_10_ or LC_25_ of papain, the levels of cytochrome oxidase subunit I (COI) and (ND1) gene expression were significantly decreased than the control group in concentration- dependent manner. The toxicity of papain might be due to its nature as a secondary metabolite that resulted in DNA damages [75, 76]. Similar results were reported by [77] who found that *C. papaya* extract caused DNA damages in exposed *B. alexandrina* snails, including the changes in number, intensity and position of DNA bands and reasoned these genotoxic to the presence of the secondary metabolites. Also, these results were in a good accordance with [59] who reported change in the level of DNA and RNA in ovotestis of *L. acuminata* snails after exposure to papain, they reasoned these damages to that papain could reduce RNA and protein content as it affected the protein bio synthesis at the transcriptional level. Also, [13] reported significant decreases in the expression level of COI and ND1 genes in *B. alexandrina* snails exposed to LC_10_ or LC_25_ concentrations of saponin. [33] reported that during the short-term tests, papain had the ability to induce lesions in DNA, which may lead to genotoxicity, cytotoxicity, or mutagenicity.

## Conclusion

This study elucidates the molluscicidal, larvicidal and genotoxicological effects of papain on *B. alexandrina* snail, free larval stages of *S. mansoni*. And hence, Papain could play an important role in the control of the intermediate snail population and ultimately elimination of schistosomiasis and decrease its transmission. The effects of field applications and the toxicity of papain to additional species are being investigated further.

## Data Availability

Any enquiries for additional information are available upon request from the corresponding author.
